# Nodular/Keloidal Scleroderma with No Systemic Involvement—A Case Report and a Review of the Literature

**DOI:** 10.3390/jcm13092662

**Published:** 2024-05-01

**Authors:** Ioana Irina Trufin, Loredana Ungureanu, Salomea-Ruth Halmágyi, Adina Patricia Apostu, Simona Corina Șenilă

**Affiliations:** 1 Clinical Hospital of Infectious Diseases, 400000 Cluj-Napoca, Romania; ioana.irina.trufin@gmail.com (I.I.T.); h_salomea@yahoo.com (S.-R.H.); adinna.apostu@yahoo.com (A.P.A.); 2 Department of Dermatology, “Iuliu Hațieganu” University of Medicine and Pharmacy, 400006 Cluj-Napoca, Romania; corina.senila@umfcluj.ro; 3 Department of Dermatology, Emergency County Hospital, 400006 Cluj-Napoca, Romania

**Keywords:** nodular scleroderma, keloidal scleroderma, nodular morphea, keloidal morphea

## Abstract

Nodular or keloidal scleroderma is a rare condition with unclear cause and sporadic mentions in the medical literature. It was first recognized in the 19th century, yet its classification is still debated due to the limited number of reported cases. This rare variant of scleroderma is associated with either progressive systemic sclerosis or localized morphea. Clinically, it presents with asymptomatic nodules or plaques, resembling spontaneous keloid formation, often found on the trunk and proximal extremities. Recent literature reviews show a predominance of women with a mean age of 44 years. Diagnosis relies on clinical and histopathological findings, which usually show overlapping features of both scleroderma and true keloids, secondarily to an excessive fibrosing reaction attributed to collagen formation. We present an unusual case of a 70-year-old female patient who displayed the coexistence of two distinct subtypes of morphea (nodular/keloidal and linear), and exclusive skin involvement, which contrasts with the typical presentation of nodular/keloidal scleroderma, often associated with organ-specific disease. However, recent publications have diverged from previous ones regarding systemic sclerosis, with no systemic involvement reported between 2018 and 2024, which we evaluated in our descriptive literature review. With less than 50 cases reported in total, our case underlines the importance of recognizing this rare disease, ensuring appropriate evaluation, treatment, and follow-up.

## 1. Introduction

Nodular or keloidal scleroderma is a rare diagnosis with uncertain pathogenesis, sporadically described in the medical literature. Its nomenclature and classification are still a subject of debate due to the paucity of reported cases; at present, nodular/keloidal scleroderma is a distinct clinical entity, associated with either progressive systemic sclerosis or localized morphea [1]. 

Clinically, it presents with asymptomatic keloid-like nodules or plaques, which are erythematous, irregularly shaped, firm at palpation, and usually affect young and middle-aged women. With a predilection for the trunk and proximal extremities, nodular/keloidal scleroderma resembles spontaneous keloid development in the context of progressive systemic sclerosis [1]. Although the pathogenetic mechanisms are still unknown, the specific skin findings arise secondarily to an excessive fibrosing reaction ascribed to collagen formation. The histopathological picture is highly variable and usually shows overlapping characteristics of both scleroderma and true keloids [2]. All these inconsistent features make not only the diagnosis, but also the subsequent therapeutic approach and patient management challenging.

We describe a case of concurrent nodular/keloidal and linear scleroderma in a 70-year-old female patient with no evidence of systemic involvement. Furthermore, we discuss the pathogenetic mechanisms, clinical and histological picture, as well as treatment options. In addition, using the PubMed database, we include and provide a descriptive review of nodular/keloidal scleroderma cases reported over the last 6 years (2018–2024), thus completing previous work on the subject, the most recent literature review on nodular/keloidal scleroderma having been published in 2020 by Richarz et al. [3], who included relevant data for the period between 1980 and 2018. Recognizing the features of this disease can help clinicians reduce misdiagnosis.

## 2. Case Report

A 70-year-old woman first presented to the Dermatology Department—Outpatient Clinic in June 2023 with a history of over 45 years of two indolent infiltrated lesions, on the left breast and on the posterior and inferior aspect of the right thigh. She also mentioned having, in the past, a similar lesion on the right shoulder, which resolved spontaneously. There was no history of trauma or exposure to radiation in the affected areas, no complaints of dry eyes or mouth, dysphagia, or morning stiffness. Her past medical history disclosed supracervical hysterectomy for fibroids, Helicobacter pylori gastritis, hypercholesterolemia, and grade 2 hemorrhoids; she had no personal or family history of keloid formation, autoimmune or connective tissue disorders, nor major internal disease. Current medications included only the use of statins (rosuvastatin).

**Figure 1:**
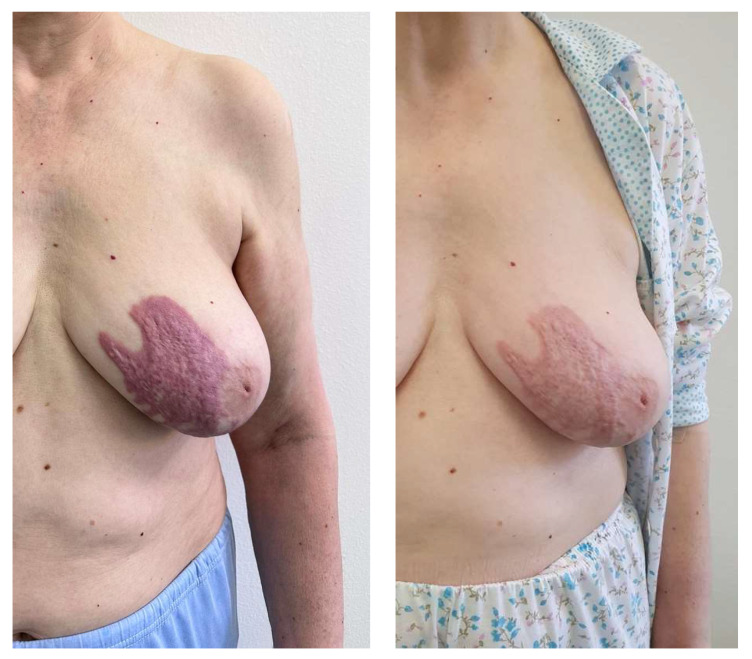
Keloidal plaque before (**left**) and after (**right**) treatment (following two 10-day-cycles of UVA phototherapy with local application of Methoxalen solution 0.05%, separated by a one-month interval with only daily topical application of potent corticosteroids).

**Figure 2:**
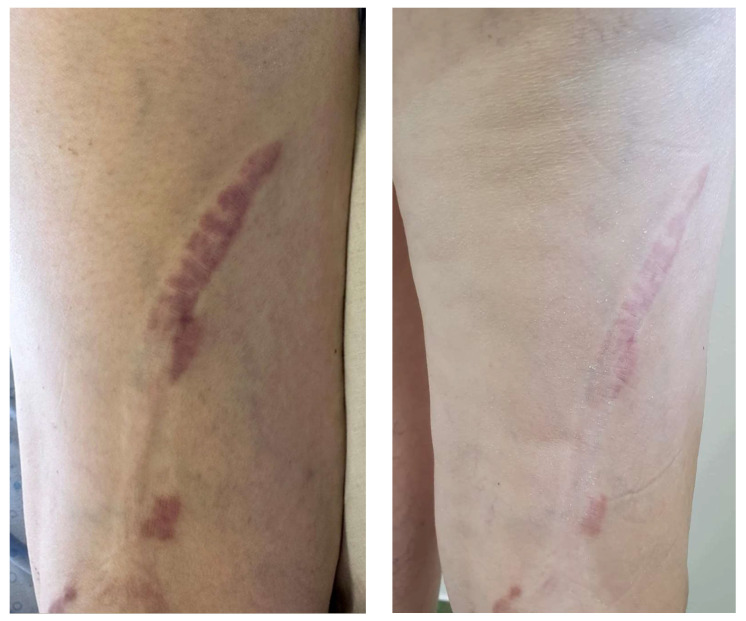
Linear plaque before (**left**) and after (**right**) treatment (following two 10-day-cycles of UVA phototherapy with local application of Methoxalen solution 0.05%, separated by a one-month interval with only daily topical application of potent corticosteroids).

Clinical examination revealed a generally good condition, with a respiratory rate of 16 bpm, blood pressure of 110/87 mmHg, and median heart rate of 60 bpm. On auscultation, a systolic murmur and arrhythmic heartbeat were identified, which prompted us to request a cardiovascular consult (detailed later). Respiratory, gastrointestinal, and renal examinations were unremarkable. The skin inspection showed a violaceous indurated keloid-like lesion on the inferior and lateral quadrant of the left breast, measuring up to 10 × 12 cm (Figure 1—left), as well as a violaceous indurated linear lesion located on the posterior and inferior side of the right thigh, with extension to the popliteal fossa and the upper aspect of the calf, with an approximate length of 17 cm (Figure 2—left). The range of motion in the knee was not limited by the presence of the skin lesion. There were no signs of mouth furrowing, oral ulcers, skin thickening, Raynaud’s phenomenon, or sclerodactyly or periungual changes.

**Figure 3:**
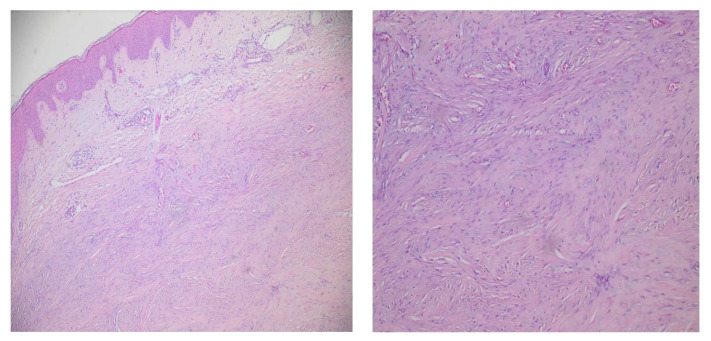
Histology of keloidal scleroderma, showing hyalinized, thickened collagen bundles and spindled fibroblasts in the reticular dermis (H&E staining, 5× magnification—**left**, and 10×—**right**).

Subsequent laboratory investigations revealed the following results: normal full blood count, electrolytes, and renal and liver function tests. Anti-Borrelia burgdorferi antibodies were not detected. Complement levels were normal. The patient had a negative antinuclear factor (ANA-antinuclear antibodies), and the panel of extractable nuclear antigen antibodies (ENAs) was negative, as well. However, the rheumatoid factor values were high (682.59 UI/mL; normal laboratory range < 14 UI/mL). Additional work-up was performed; hand radiographs revealed symmetrically reduced mineral density, as well as minimal narrowing of the distal and proximal interphalangeal joint spaces of both hands, with normal joint alignment. Chest X-ray examination revealed a discrete reticulonodular pattern involving the medial (hilar) aspects and external supradiaphragmatic peripheries of each lung, apical and bilateral pachypleuritis, as well as calcifications of the aortic arch. A pulmonary consult was solicited, to rule out sarcoidosis, due to the frequent association of various isolated violaceous skin lesions with lung involvement. Additionally, angiotensin-converting enzyme (ACE) levels were within normal range. Based on clinical, radiologic and laboratory findings, the diagnosis of sarcoidosis was improbable. As previously mentioned, a cardiovascular consult was also requested; the echocardiogram identified grade 2 mitral and tricuspid insufficiencies, mild pulmonary hypertension, as well as intermittent arrhythmic contractions. The ECG Holter monitoring confirmed the diagnosis of atrial fibrillation and the patient started anticoagulant and antiarrhythmic medication. Other investigations included mammography and breast ultrasound, which showed no abnormalities. Skin ultrasound of the keloidal lesion described local changes in the epidermis and dermis, with the presence of hyperechoic nodular areas alternating with hypoechoic ones, especially in the superficial dermis, and focal extensions to the deeper dermis, extending variably between 3 and 6 mm. The epidermis was irregularly thickened, while the dermo–epidermal junction was doubled by a hyperechoic band throughout the lesion. On Doppler ultrasound, focal areas of hyperemia were identified. Biopsy specimens taken from the keloidal lesion showed a normal epidermis; the dermis, however, was significantly thickened by a fibrous proliferation, consisting of eosinophilic collagen fibers in a linear pattern and spindled fibroblasts (Figure 3). The histological evaluation of the subcutaneous adipose layer did not show any alterations.

The clinical and histological findings were consistent with a diagnosis of nodular/keloidal scleroderma. Due to the frequent association with progressive systemic sclerosis, the patient was then referred to a rheumatologist. The rheumatological evaluation could not find clinical signs of calcinosis, sclerodactyly, or telangiectasia; additionally, the nail fold microscopy showed no alterations of the capillary loops. Having already performed organ-directed tests and consultations (cardiovascular, pneumology, chest, and hand radiographs), the rheumatology report did not find sufficient evidence in favor of organ involvement, therefore a diagnosis of systemic sclerosis was excluded.

We started topical treatment with clobetasol propionate 0.05% ointment, in association with daily UVA phototherapy after local application of Methoxalen 0.05% solution in two cycles of 10 days each, with favorable results (Figure 1—right and Figure 2—right). The patient was admitted to our clinic on two separate occasions at one month interval. The topical application of corticosteroids was then gradually tapered over 2–3 months. Intralesional corticosteroids were also discussed as a treatment option, but the patient deferred further treatment because of the sufficient improvement under phototherapy. The lesions have remained stable on following consults; however, close observation and monitoring for any progression of the disease are taken forward.

## 3. Discussion

Scleroderma or systemic sclerosis is a challenging and relatively rare clinical condition of autoimmune origin and undetermined etiology. Excessive collagen formation may affect the skin in a localized or generalized fashion, but it can also lead to fibrosis of internal organs, the lungs and esophagus being most often affected [1]. Alternate nomenclature includes scleroderma or morphea, which are the terms used to describe the exclusive skin involvement. On the other hand, systemic scleroderma, or progressive systemic sclerosis, implies the involvement of internal organs and follows a chronic, inflammatory course, with organ-specific symptoms. Raynaud’s phenomenon is reported to be the first and most common finding and may precede the onset of the disease by years [1].

Cutaneous findings show high clinical variability; nonetheless, the histopathological clues are identical and essential for diagnosis [2]. Among the multiple types of lesions, the keloidal or nodular variant is considered the rarest form [2], and it is the result of abundant collagen deposition and thickening of the dermis.

Elevated lesions in the context of cutaneous scleroderma have first been described in 1854 by Addison, as ‘untrue keloid’, followed four decades later by Unna, who indicated their resemblance with keloid scars, and named this clinical subtype of scleroderma “keloid-like scleroderma” [4]. Various cases have been reported in the following years, using a descriptive terminology. In 1954, Korting stated that the raised lesions (whether nodular, keloidal, or distinctively shaped) have identical pathological backgrounds and diverge only in means of clinical picture [4]. Subsequently, the terms ‘nodular’ and ‘keloidal’ scleroderma have been used synonymously in the medical literature.

The classification of keloidal/nodular scleroderma into a separate entity is still a subject of debate; it was initially considered to be a subtype of localized scleroderma, yet most cases seem to be associated with systemic sclerosis and visceral involvement [3]. It has been reported that skin lesions usually arise in the first six months after the diagnosis of systemic sclerosis, during a time of clinically active disease, but they can also precede and anticipate its onset [5]. Although uncommon, nodular/keloidal scleroderma can occur alone, with exclusive skin involvement and no evidence of systemic disease, as a variant of localized scleroderma or morphea. The first reported case of keloidal scleroderma alone (skin limited) dates to 1985, when Akintewe et al. [6] described a 17-year-old Nigerian woman presenting with generalized pruritic and tender hyperpigmented keloids and thickening of the skin with no definite involvement of internal organs.

For this reason, some authors argue that the term ‘morphea’ should be used with only limited skin disease, while ‘scleroderma’ implies the presence of systemic/multiorgan disease [2]. However, this suggested terminology is not largely used since diagnostic criteria and a consensus are still expected.

With only a few published cases (epidemiological data based on case reports), and indefinite standardization in terms of nomenclature, lack of clinical or histological diagnostic criteria, its incidence, geographic distribution, or other epidemiological parameters are difficult to determine. There is no predominance of any age group or sex, although some publications mention a predilection for middle-aged women [3,5]; most of the reported cases fall into the 11–71 years age interval at presentation [7].

In 2020, Richarz et al. [3] published the most recent (to date) literature review on the association of keloidal or nodular scleroderma and systemic sclerosis, searching for the frequency of general symptoms and organ involvement. A total of 27 articles published between 1980 and 2018 fulfilled the inclusion criteria of the systematic review. They identified a total of 37 cases of nodular/keloidal scleroderma and systemic sclerosis, and, together with 4 cases from their institution, a total of 41 patients were assessed.

The following results were reported: 82% of patients were women, with a mean age of 44 years. The most frequently reported systemic symptoms were sclerodactyly (97.6%), followed by Raynaud phenomenon (75.6%), with arthralgia and arthritis occurring in fewer cases (34.1% and 7.3%, respectively). Regarding laboratory findings, ANA testing was reported in 37 of the 41 cases and the speckled pattern was recurring (43.2%, 16/37). More specific antibodies for systemic sclerosis (anti-Scl70, anti-centromere, and anti-U3-RNP) were investigated in 33 of the 41 cases, and were positive only in 13 (39.4%), with anti-Scl70 the most common antibody (21.2%, 7/33). Evaluation of visceral involvement showed that the lungs were affected in 46.3% (19 of 41) cases, followed by oesophageal dysmotility or gastro-oesophageal reflux disease in 41.5% (17) of patients, and unspecified renal disfunction in 5 patients (12.2%).

The authors then compared these results with the distribution of organ involvement in both diffuse and localized systemic sclerosis, noting that in nodular/keloidal scleroderma, joint and esophageal involvement was present in less than half of the patients (34.1% and 41.5%, respectively), compared to the higher frequencies of arthralgia (98% in diffuse systemic sclerosis and 90% in limited systemic sclerosis) and esophageal dysmotility, which is the most commonly reported organ dysfunction, affecting 90% of patients with diffuse and 80% of patients with limited systemic sclerosis, followed by interstitial lung disease (70% in diffuse systemic sclerosis and 35% in the limited form). The differences in the distribution of organ involvement were most likely caused by the small number of cases.

Using the PubMed database, we continued the research of Richarz et al. by performing a descriptive literature review of the cases reported over the last 6 years (2018–2024), applying keywords specific for alternate nomenclature: ‘keloidal scleroderma’, ‘keloidal morphea’, ‘nodular scleroderma’, and ‘nodular morphea’. Selection criteria included articles written in English and the presence of histological confirmation of the diagnosis. All patients exhibited characteristic skin lesions and a potential systemic involvement was evaluated. We identified four case reports [1,8,9,10], and together with the case from our institution, a total number of five patients with nodular/keloidal scleroderma. Significant clinical and serological information is summarized in Table 1.

In our review, all patients were women with age intervals ranging from 50 to 70 (our case) years; ethnic origin was not specified in most cases. Apart from trimethoprim/sulfamethoxazole reported in a single case prior to the development of keloidal lesions, there were no other mentions of potential trigger factors. The skin lesions were mainly located on the thorax and on the proximal parts of the upper limbs, following the typical distributions reported. Some remarkable findings were the absence of sufficient criteria for a diagnosis of progressive systemic sclerosis; in other words, almost all cases of nodular/keloidal scleroderma were limited to the skin since there were no signs of organ involvement.

This aspect is highly contrasting to previous data regarding the more frequent connection to systemic disease. Moreover, the laboratory tests showed only altered titers of the antinuclear antibodies, while specific antibodies for systemic sclerosis (anti-Scl70, anti-centromere, and anti-U3-RNP) were either negative or uninvestigated. Overall, the treatment options showed poor results. However, due to the lack of complete clinical information and follow-up, no conclusions can be drawn from the information provided.

Concurrent and distinct nodular/keloidal and linear sclerodermic skin changes have not been previously described. We found two cases of nodular lesions developing within areas of linear morphea [11,12], and two cases of multiple nodular sclerotic lesions following a linear distribution [13,14], but the coexistence of nodular/keloidal and linear sclerotic lesions arising distinctively in separate body regions has not been reported. Jain K et al. [12] suggested an underlying genetic mosaicism, since their patient had a linear plaque that had developed along the Blaschko lines on the lower extremity; nonetheless, the dermatomal distribution is controversial, and the other reported case of nodules developing on linear sclerotic plaques [11] did not follow a blaschkoid pattern.

## 4. Etiopathogenesis

The etiology and pathogenesis of nodular scleroderma need a better understanding. Although nodular/keloidal lesions almost always arise in patients with a previous diagnosis of systemic sclerosis, they are not strictly related and exclusive to an ongoing course of progressive disease; in some cases, the keloid-like lesions can be the only clinical finding, with no other clues (clinical, laboratory) in favor of associated scleroderma [4]. Earlier reports outline different views on the matter. James et al. [15] argue that the development of keloidal lesions is simply a reaction to the inflammation in scleroderma affecting patients with a genetic predisposition. Mizutani et al. [16] address a different perspective and note that sclerodermic lesions in the active inflammatory phase set the profibrotic grounds for the emergence and progression of keloid reactions, as a response to traumatic events. Sclerodermic skin in the early inflammatory stages primes a potential fibrosing process, setting a background of overexpressed mediators that support the keloid-like reaction [16].

A widely accepted hypothesis on pathogenesis states that the development of hypertrophic lesions may be favored by some trigger factors, such as local trauma, and the inherent inflammation of scleroderma in patients prone to keloid formation. There is also a remark that excessive fibrosis typically occurs in areas affected by scratching (mechanical factor) and with a known predilection to the development of keloids, such as the neck, trunk, upper arms, and legs [4]. A literature review by Kassira et al. [17], published in 2015, noted that ten percent of the included cases had a trigger factor prior to the development of keloidal lesions, such as infection, tetanus vaccine, or D-penicillamine. Some reports have speculated a potential pathogenetic role of acid-fast bacteria (AFB), hepatitis C virus (HCV), or organic solvents [18,19]. In 2005, Melani et al. [20] described a case of nodular scleroderma with simultaneous mild HCV; based on other previously published studies on the association between HCV and other immunomodulated disorders, they argued that chronic HCV infection may be a trigger factor to an autoimmune process.

Immunological mechanisms may also be responsible for fibrosis induction and collagen synthesis [21]. It has been demonstrated that the extracellular matrix proteins have an increased expression, with immunohistochemical studies focusing on fibronectin and tenascin [4]. In fibrosing processes (both hypertrophic scars and keloids), their expression is directly related to the intensity of inflammation; nonetheless, in systemic sclerosis this connection follows a different course. The expression of tenascin (which is also a marker for tissue remodeling, strongly expressed in areas of wound healing and tumor invasion) is more intense and persists even after the remission of the inflammatory phase [16].

Furthermore, fibrogenic cytokines, such as transforming growth factor (TGF)-beta, connective tissue growth factors (CTGFs), and platelet-derived growth factor (PDGF) are over-expressed in nodular/keloidal scleroderma lesions and are involved in their induction [1,7]. TGF-beta plays an important role in collagen synthesis, through various mechanisms, such as stimulation of fibroblast proliferation and chemotactic activity of macrophages, as well as downregulation of matrix metalloproteinases in association with upregulation of proteinase inhibitors [22]. All these processes consequently converge to an increase in extracellular matrix (ECM) production [23]. The connective tissue growth factor (CTGF) has more recently been suggested to contribute to dermal fibrosis; its synthesis is induced in fibroblasts or mesenchymally derived cells after activation by TGF-beta, and it is found to upregulate collagen, fibronectin, and integrin expression. Apart from being detected in skin specimens of localized scleroderma, higher levels of CTGF mRNA have been reported in the sclerotic stage rather than the inflammatory stage. This suggests the implication of CTGF in the maintenance of already formed lesions and not in their induction [22]. The cartilage oligomeric matrix protein (COMP), type XII collagen, and fibrillin-1 also seem to contribute to the pathogenesis of fibrosis; high COMP expression has been demonstrated in keloid, while in scleroderma lesions it promotes fibrosis through stimulation of fibroblast activity and modulation of the dermal collagen network [5].

Epigenetics may play an important role in collagen expression. In 2012, Etoh et al. [24] published an in-depth study on the implications of epigenetics in tissue fibrosis, with a focus on scleroderma. They found that cultured dermal fibroblasts from sclerodermatous lesions showed an upregulation of type I collagen, the most abundant ECM component in the skin; similarly, its expression was upregulated in keloids. However, the specific mechanism has not yet been formulated. Epigenetics have recently provided insight into the potential implications of microRNAs (miRNAs) in the pathogenesis of skin fibrosis. Briefly, miRNAs are non-coding RNAs that bind to untranslated regions (UTRs) of mRNAs, thus inhibiting their translation into proteins. More than 1000 miRNAs have been identified and they are considered important regulators of cell development and differentiation, growth control, and apoptosis [25]. Etoh et al. [24] found that several miRNAs are suppressed both in sclerodermic and keloid lesions compared to normal skin; among these, the downregulation of miR-7 is correlated to the overexpression of alpha2(I) collagen in dermal fibroblasts and may be an important marker of tissue fibrosis. It is postulated that the administration of microRNA has a protective role against fibrosis and may also be able to reverse it [25].

## 5. Clinical Picture

Elevated sclerotic plaques are the hallmark of this condition. The diversity of clinical manifestations relies on aspects such as shape, dimensions, number of lesions, and their distribution. Labandeira et al. [4] identified two main categories of skin findings: the first were hemispherical papules 2–30 mm in diameter and the second type were plaque-like lesions, adjusted by the following factors—their vertical level, the occurrence on normal or previously sclerotic skin, their monomorphic or polymorphic appearance, and the association with other specific features of systemic sclerosis.

Typically, at presentation, the lesions present as skin-colored nodules or plaques, which are firm at palpation, non-tender, and have irregular margins and diameters ranging from 2 mm to 3 cm, affecting specific areas. The most frequently described sites are, in order of decreasing frequency: the trunk, arms, shoulders, neck, upper legs, gluteal site, ears, nose, and face [7]. The lesions may arise on normal-looking or on already affected (sclerotic) skin and can be isolated, generalized, or, in rare cases, linear [2,3,4,13,14]. There are descriptions of indolent linear infiltrative plaques with unusual distribution (lower abdomen) and a histological picture compatible with the diagnosis of nodular/keloidal scleroderma [13]. A blaschkoid distribution has been reported in a singular case, different than the linear pattern, which is not dermatomal and does not follow the Blaschko lines [12]. Jain et al. [12] describe a case of Blaschko linear scleroderma of the lower limb with nodules arising on sclerodermatous skin, suggesting that a genetic mosaicism can lead to the formation of skin cell clones (including fibroblasts) distributed in a dermatomal pattern.

Although there is a noticeable variety in the clinical picture, most cases show multiple papular lesions in areas prone to keloid formation, such as the neck, trunk, upper arms, and legs [4]. In some cases, they mimic the morphology of keloids, with a firm consistency, irregular surface, and arciform margins, which can extend with pseudopod-like projections [9]. However, unlike true keloids, the lesions in nodular/keloidal scleroderma are generally asymptomatic, with pruritus reported only in a small number of cases [4]. Keloids are the main differential diagnosis, followed by dermatomyofibroma, fibromatosis (desmoid tumors) [2,3], and cutaneous mucinosis, which is a clinical condition that may also coexist with systemic sclerosis or morphea [5].

Briefly, the clinical diagnosis is based on the previously detailed skin findings, arising either de novo, or on already formed sclerotic plaques, in patients with no history of trauma/radiation or keloid formation. A biopsy with histological evaluation of the specimen lead to the final diagnosis. The occurrence of raised lesions extends over the first year of systemic sclerosis [3], therefore their presence might be an indicator of systemic disease and should prompt physicians to consider other criteria for an early diagnosis.

## 6. Histology

The histological picture may show features specific for either scleroderma or keloid/hypertrophic scarring; there are also situations in which both scleroderma and keloid-type findings can be present in the same biopsy, or they follow one another in evolution [4]. In other words, the biopsy reports may outline: (1) the characteristics of keloid of hypertrophic scarring, (2) the characteristics of scleroderma, (3) the characteristics of both keloid and morphea type in the same biopsy specimen, (4) scleroderma-like characteristics initially, followed by the subsequent development of keloid-type characteristics [22]. Ohata et al. [26] recognized the confusion and tried to classify the histopathological changes by dividing them into two categories: genuine morphea type and scar-keloid type. They reviewed the cases to date and compared the descriptions of collagen, fibroblasts, and vascularity, stating that in the genuine morphea type there is an arrangement of thickened collagen fibers in the reticular dermis and occasionally in the papillary dermis, in contrast to the scar-keloid type of nodular/keloidal morphea, which has a more unsystematic collagen distribution, with either thin bundles in a parallel orientation with plump fibroblasts and dilated venules (in scars) or thick eosinophilic fibers disorderly arranged among fibroblasts and reduced vascularity (in keloid). The authors also reported, in the literature review they performed, that nineteen cases (including their own) of NM/KM showed histological features of genuine morphea and fifteen NM/KM cases were classified as scar-keloid type.

Histological findings exhibit an endo-exophytic growth determined by a mixture of dermal sclerosis with eosinophilic or glassy collagen. The hyalinized or homogenized collagen fibers may be oriented in whorl-like patterns in hypocellular areas (keloid-specific) or run parallel to the epidermis (sclerodermatous features); the center of the lesion may also include fragmented elastic fibers [2,8,9]. The diagnosis is supported by the presence of keloidal collagen aggregations (bright eosinophilic bundles) and a variable number of fibroblasts and/or myofibroblasts on a background of classical scleroderma (thickened hypocellular dermis with swollen collagen bundles) [9]. In rare cases, there are also histological descriptions of focal groups of spindle cells and lymphoplasmacytic inflammatory infiltrates, the latter being described in more recent lesions as small perivascular and periadnexal cellular groups [2,8,9].

The diagnosis can therefore be challenging since the frequently overlapping features require the exclusion of conditions such as hypertrophic scars, keloids, fibromatosis, dermatomyofibroma, desmoid tumors, and scleromyxedema [2,3,8]. Spontaneous keloids have a racial or familial predilection; dermatomyofibroma occurs more commonly in young women and shows a dermal proliferation of fibroblasts and myofibroblasts, while desmoid tumors consist mostly of myofibroblast proliferation, with infiltrative growth pattern and potentially aggressive behavior [2]. The presence of spindle-shaped elements with atypical nuclei should be differentiated from mesenchymal tumors that may mimic keloidal overgrowths. Castelli et al. [9] elaborated, in their study, a comprehensive approach to the histological picture and the distinctive features, as well as immunohistochemical patterns not only for nodular/keloidal scleroderma but also for the similar clinical conditions that need to be excluded.

The complex interaction between cellular elements, proteins of the extracellular matrix, cytokines, and genetic and local factors create both the clinical and histological picture.

## 7. Treatment

There is no consensus on the therapeutic options in nodular/keloidal scleroderma, as they are mostly based on case reports, the available data being inconclusive. The condition seems to be refractory to therapy and the attempted therapies show variable responses. There are no reports of spontaneous resolution.

Currently, the treatment options available for nodular/keloidal scleroderma include the topical or intralesional use of corticosteroids, topical or oral vitamin D analogs, topical tacrolimus or imiquimod, UVA1, PUVA, systemic steroids, doxycycline, NSAIDs, azathioprine, cyclosporine, and methotrexate (in association or not with pulsed methylprednisolone) [1,5,7,17,23].

The literature review conducted by Kassira et al. (published in 2015) [17] noted an overall lack of response following treatment with local and/or systemic corticosteroids, with only three cases, out of the 22 included, showing partial response to treatment, and one case with complete response to systemic steroids. The use of D-penicillamine is controversial, as there are reports showing both complete resolution and progression under therapy. There are also data on complete resolution following surgical removal of lesions [1] and one study reported complete resolution following extracorporeal photochemotherapy [4].

New therapeutic options using potential antifibrotic agents, such as an antisense oligonucleotide to CTGF (EXC001) and a small interfering RNA to CTGF (RXI-109) are under investigation in clinical trials. Additionally, in more recent studies, a microRNA-29 mimic shows promising results in animal models and clinical trials [1]. Soria et al. [27] conducted a preclinical study evaluating the effects of imatinib (Glivec RRRR) on scleroderma and normal dermal fibroblasts; they demonstrated a decreased proliferation of in vitro fibroblasts from patients with both localized and systemic scleroderma—nonetheless, further clinical studies are required.

## 8. Conclusions

We describe an unusual case of nodular/keloidal and linear scleroderma in a 70-year-old female patient with no other signs of systemic involvement. This is a contrasting clinical picture, different from previous reports since most nodular/keloidal scleroderma patients have organ-specific disease with earlier onset (middle-aged women). The long history of two indolent skin lesions arising de novo, in the absence of potential trigger factors, such as trauma or exposure to radiation on those sites are important diagnostic clues. Furthermore, the evaluation for internal disease could not meet the criteria for systemic sclerosis or other connective tissue disorders. Apart from the exclusive skin involvement with histological confirmation of nodular/keloidal morphea, other remarkable findings are the high levels of the rheumatoid factor, contrasting to a normal serological/immunological picture.

The most recent publications are in incongruity with previous ones in terms of association with systemic disease. There was no systemic involvement found in the selected cases reported between 2018 and 2024, and with less than 50 cases in total found in the medical literature, the presented case adds to existing knowledge given the rarity of the condition, underlining the importance of its recognition and differentiation from conditions such as simple keloids or hypertrophic scars, so that an early systemic process would not be missed. The connections between the clinical and histological picture help establish the correct diagnosis, ensuring adequate evaluation, treatment, and follow-up. However, further research is required to gain a deeper understanding of pathogenesis and treatment.

## Figures and Tables

**Table 1:** Demographic characteristics and clinical and serological features of patients with nodular/keloidal scleroderma between 2018 and 2024.

First Author/Year [Ref.]	Yu D/2020 [1]	Li Y/2023 [8]	Castelli E/2020 [9]	Dadkhahfar S/2019 [10]	Our Case
Sex	F	F	F	F	F
Age (years)	34	60	50	54	70
Ethnicity	ND	ND	Caucasian	ND	Caucasian
External Trigger	trimethoprim/sulfamethoxazole	ND	ND	ND	patient denies
Distribution of lesions	arms, chest, abdomen, flank	arms, anterior chest, abdomen	sternum, pectoral regions, shoulders	trunk and thighs	left breast
Organ involvement/Systemic symptoms	Raynaud’s phenomenon	abnormal nail fold capillaroscopy, pulmonary cysts and interstitial thickening	no organ involvement	no organ involvement	no organ involvement
Positive laboratory tests	ANA (>1:1280)	ANA (1:320)	unremarkable	unremarkable	elevated rheumatoid factor
Diagnosis	morphea–skin limited	potential systemic involvement	morphea–skin limited	morphea–skin limited	morphea–skin limited
Other relevant data	annular plaques	-	-	new nodular lesions occurring under systemic treatment	association with a linear sclerodermic plaque
Treatment	systemic steroids, topical clobetasol 0.05% ointment	methotrexate 7.5 mg weekly	ND	pulsed intravenous high-dose methylprednisolone (1000 mg; three consecutive days monthly for six months) combined with oral methotrexate (15 mg weekly). cyclosporine; 300 mg daily intralesional triamcinolone	topical clobetasol 0.05% ointment in association with daily UVA phototherapy with meladinine 0.05%
Results/Duration of lesions	Improvement	ND	ND	mild improvements in skin induration six months after the end of methylprednisolone pulses and methotrexate. marked improvement of skin induration following treatment with cyclosporine. overall unsatisfactory results.	significant improvement

F—female; ref.—reference; ND—no data; ANA—anti-nuclear antibodies.

## Data Availability

Not applicable.
